# Association of Sleep Electroencephalography-Based Brain Age Index With Dementia

**DOI:** 10.1001/jamanetworkopen.2020.17357

**Published:** 2020-09-28

**Authors:** Elissa Ye, Haoqi Sun, Michael J. Leone, Luis Paixao, Robert J. Thomas, Alice D. Lam, M. Brandon Westover

**Affiliations:** 1 Department of Neurology, Massachusetts General Hospital, Boston; 2 Division of Pulmonary, Critical Care and Sleep, Department of Medicine, Beth Israel Deaconess Medical Center, Boston, Massachusetts

## Abstract

**Importance:**

Dementia is an increasing cause of disability and loss of independence in the elderly population yet remains largely underdiagnosed. A biomarker for dementia that can identify individuals with or at risk for developing dementia may help close this diagnostic gap.

**Objective:**

To investigate the association between a sleep electroencephalography-based brain age index (BAI), the difference between chronological age and brain age estimated using the sleep electroencephalogram, and dementia.

**Design, Setting, and Participants:**

In this retrospective cross-sectional study of 9834 polysomnograms, BAI was computed among individuals with previously determined dementia, mild cognitive impairment (MCI), or cognitive symptoms but no diagnosis of MCI or dementia, and among healthy individuals without dementia from August 22, 2008, to June 4, 2018. Data were analyzed from November 15, 2018, to June 24, 2020.

**Exposure:**

Dementia, MCI, and dementia-related symptoms, such as cognitive change and memory impairment.

**Main Outcomes and Measures:**

The outcome measures were the trend in BAI when moving from groups ranging from healthy, to symptomatic, to MCI, to dementia and pairwise comparisons of BAI among these groups.

**Findings:**

A total of 5144 sleep studies were included in BAI examinations. Patients in these studies had a median (interquartile range) age of 54 (43-65) years, and 3026 (59%) were men. The patients included 88 with dementia, 44 with MCI, 1075 who were symptomatic, and 2336 without dementia. There was a monotonic increase in mean (SE) BAI from the nondementia group to the dementia group (nondementia: 0.20 [0.42]; symptomatic: 0.58 [0.41]; MCI: 1.65 [1.20]; dementia: 4.18 [1.02]; *P* < .001).

**Conclusions and Relevance:**

These findings suggest that a sleep-state electroencephalography-based BAI shows promise as a biomarker associated with progressive brain processes that ultimately result in dementia.

## Key Points

**Question:**

Can an electroencephalography-based brain age index, calculated as the difference between chronological and estimated brain age through sleep electroencephalography, serve as a potential biomarker associated with dementia?

**Findings:**

This cross-sectional study of 9834 polysomnograms using machine learning found that brain age index increased monotonically from nondementia to dementia subpopulations.

**Meaning:**

These findings suggest that brain age index may be a useful biomarker associated with dementia and could have utility as a screening tool for the presence of underlying neurodegenerative disease and monitoring of disease progression.

## Teaser

This cross-sectional study examines a sleep electroencephalography-based brain age index as a potential biomarker associated with dementia.

## Introduction

Dementia is an increasing cause of disability and loss of independence in the elderly population. After age 65 years, the prevalence of dementia increases 2-fold every 5 years.^1^ Nevertheless, dementia remains largely underdiagnosed.^2^ Biomarkers that use clinical testing commonly performed in elderly patients could help close this diagnostic gap. While electroencephalography (EEG) has been identified as a potential biomarker, its use in dementia screening is not yet a part of clinical practice.^3^

Sleep undergoes changes with age, reflected in the EEG. With aging, sleep becomes fragmented, and the proportion of stage 1 sleep increases while slow-wave sleep (SWS) decreases.^4^ With neurodegenerative diseases, sleep becomes more fragmented, shows reduced slow-wave sleep and rapid eye movement (REM) sleep, and sleep spindles and vertex waves become less well-formed and less numerous.^4^

We recently developed the brain age index^5^ (BAI), a machine learning model that estimates the difference between the computed brain age based on sleep EEG and chronological age. Increased BAI signifies deviation from brain aging within reference ranges; thus, it is natural to expect that BAI may reflect the presence and severity of dementia. While other approaches to estimating brain age are based on magnetic resonance imaging (MRI)^6^ and awake EEG,^7^ our BAI is uniquely based on the sleep EEG. The gap between MRI-based brain age and chronological age is associated with risk of dementia^8^ and conversion of mild cognitive impairment (MCI) to dementia.^9^ Our BAI also has been associated with neurological and psychiatric disease, hypertension, diabetes, and mortality.^10^

Here, we investigated the association of sleep EEG-based BAI and dementia. We also compared BAI of patients with MCI, an intermediate state between unimpaired cognition and dementia with objective cognitive impairments but largely preserved functional capabilities,^1^ and BAI of patients with cognitive symptoms but no diagnosis of dementia or MCI (eg, due to nutritional deficiencies, psychiatric disorders, or other conditions). Finally, we investigated the association of BAI with neuropsychological scores, the contribution of dementia on increased BAI compared with other covariates, and correlations between dementia and EEG features in the brain age algorithm.

## Methods

The Partners HealthCare institutional review board approved all study procedures and waived the requirement for informed consent for this retrospective data analysis because data were deidentified. This study is reported following the Strengthening the Reporting of Observational Studies in Epidemiology (STROBE) reporting guideline.

### Data set

This cross-sectional study retrospectively analyzed a data set of polysomnograms acquired in the Sleep Laboratory at Massachusetts General Hospital from 2009 to 2017, which included 9834 polysomnograms. Polysomnograms were recorded adhering to the American Academy of Sleep Medicine standards. The data set contains 3 types of sleep tests: diagnostic, full-night continuous positive airway pressure, and split-night continuous positive airway pressure. Polysomnograms were clinically annotated in 30-second epochs according to American Academy of Sleep Medicine standards as wake, non-REM stage 1 (N1), non-REM stage 2 (N2), non-REM stage 3 (N3), and REM. The data set is summarized in eTable 1 in the Supplement.

### Calculation of the BAI

The brain age model is a generalized linear model that uses a softplus function as the link function, which is optimized to estimate an individual’s age based on an overnight EEG. As defined in Sun et al,^5^ a variety of EEG features were extracted. Features included spectral powers (absolute and relative or ratios), and measures of signal complexity. More technical details are provided in the eAppendix in the Supplement.

### Clinical Data Extraction

Clinical data (eg, demographic characteristics, diagnoses, medications, problem lists, and clinical notes) were extracted from questionnaires completed before the sleep study and from electronic medical records. Scores for the Mini-Mental State Examination (MMSE)^11^ and Montreal Cognitive Assessment (MoCA),^12^ when available, were extracted from clinical notes before or within 1 year after the sleep study using regular expressions. These examinations were performed owing to concern for cognitive problems as part of clinical care and often recorded within neuropsychiatric evaluations.

### Assigning Patients Into Dementia, MCI, Symptomatic, Nondementia, and Healthy Groups

Patients were categorized into dementia, MCI, symptomatic, nondementia, and healthy groups. These groups were defined using formal inclusion and exclusion criteria listed in the Table and were determined by natural language processing applied to the electronic medical record, using custom software. A healthy group was included as a subset of the nondementia group, defined as individuals with no prior history of neurologic or psychiatric disease. Notably, in our institution’s electronic medical record, problem lists are lists of active, reoccurring medical problems for each patient, whereas encounter diagnoses are often preliminary diagnoses that physicians code for each patient encounter. To ensure the authenticity of our dementia and MCI groups, we used the problem list as a criterium because it is a more reliable source of diagnosis than only encounter diagnoses. We used encounter diagnosis as a criterium for the symptomatic group to make the symptomatic group inclusive of any patients with potential cognitive concerns and the nondementia group exclusively asymptomatic (ie, the nondementia group was obtained by excluding the aforementioned groups).

**Table.** Definitions of Dementia, MCI, Symptomatic, Nondementia, and Healthy Groups and Exclusion Criteria

Group	Criteria^a^	No.	
		Studies	Patients
Dementia	Using ≥1 dementia-related medication with a diagnosis containing any dementia keyword^b^^,^^c^ Diagnosis in the problem list containing any dementia keyword^c^ MoCA score ≤19 and no MoCA scores >27 after the sleep study MMSE score ≤25	96	81
MCI	Diagnosis in the problem list containing any MCI keyword^d^ MoCA score 20-25 and no MoCA scores >27 after the sleep study	55	44
Symptomatic	Diagnosis containing any dementia or dementia-related keyword in the encounter diagnosis, problem list, and/or medical history^e^	1361	1075
Nondementia	Does not belong to the dementia, MCI, or symptomatic group but may have a prior history of neurological or psychiatric disease in an encounter diagnosis, problem list, and/or medical history^f^	3632 studies	3024 patients
Healthy	Subset of the nondementia group with no history of neurological or psychiatric disease in encounter diagnosis, problem list, and/or medical history^f^	2799	2336
Excluded	Age <50 y Received a diagnosis for developmental delay, brain tumor, or neoplasm Received a diagnosis for stroke, brain injury or trauma, or seizure before sleep study	4690	4053

Abbreviations: MCI, mild cognitive impairment; MMSE, Mini-Mental State Examination; MoCA, Montreal Cognitive Assessment.

^a^ Inclusion criteria are based on data entered in the medical record before the sleep study or at most 1 year after the sleep study unless otherwise stated. A patient was in a group if they met at least 1 of the inclusion criteria and did not meet any of the exclusion criteria. All groups except the healthy group were mutually exclusive. The healthy group was a subset of the nondementia group.

^b^ Dementia-related medications include aricept, donepezil, exelon, rivastigmine, memantine, namenda, namzaric, razadyne, and galantamine.

^c^ Dementia keywords were *dementia* and *Alzheimer*.

^d^ MCI keywords were *MCI*, *mild cognitive impairment*, and *minimal cognitive impairment*.

^e^ Dementia-related keywords were *cognitive*, *memory*, *amnesia*, *agnosia*, *apraxia*, *aphasia*, and *mental*.

^f^ Neurological or psychiatric disease included cerebral hemorrhage, Parkinsonism, mood disorder, psychotic disorder, intracranial hypertension, cerebral palsy, epilepsy, hydrocephalus, encephalopathy, multiple sclerosis, and delirium.

### Matching Between Groups

To isolate the association of dementia with BAI, we matched patients in each group by sex, age, and sleep study type, which are potential confounding variables. Older age may increase risk of dementia and lower BAI owing to systematic biases,^13,14^ while age-specific, cognitive, and structural changes in the brain may be more profound in men than women.^15,16^ Sleep study type could also be associated with different sleep disturbances that may confound brain age and dementia risk.^4^ We use matching as a nonparametric method for adjusting these confounding variables. The dementia group served as the reference for matching. For each patient in the dementia group, we found matching individuals in each other group, for whom sex and sleep study type were exact matches and age fell within 5 years. We sampled studies from each of the groups to generate a maximum subsample with the same distribution of covariates as that of the dementia group; the MCI group was oversampled with replacement owing to its smaller size.

### Spectrograms and Hypnograms

We selected representative spectrograms and hypnograms for each group. We first standardized the brain age features, then computed the mean brain age features for each group. For each group, the 30 participants closest to the group mean (based on Euclidean distance) were selected, and the participant with the most visually representative spectral features (ie, amount of spindle band power and peak frequency of α oscillation) was selected manually. We also ensured that the representative spectrograms had similar hypnogram structures.

### Statistical Analysis

To investigate the association between BAI and dementia group membership, a Cuzick test for trend^17^ was computed to measure the trend of BAI across groups, ordered as (1) dementia, (2) MCI, (3) symptomatic, and (4) nondementia. The healthy group is excluded from trend analysis owing to being a subset of the nondementia group. Pairwise comparisons of BAI among all groups were conducted using independent samples *t* tests. To check for differences in proportions of potentially confounding medical and psychiatric conditions between the matched dementia vs nondementia groups, we performed 2-sample proportion *z* tests comparing the prevalence of 9 prespecified conditions between the dementia and nondementia groups: smoking, alcoholism, insomnia, mood disorders, psychotic disorders, anxiety disorders, cardiovascular diseases, diabetes, and obesity. Additionally, we examined the weight of BAI in association with sleep macrostructure features and sleep fragmentation index in estimating presence of dementia based on matched groups by building a logistic regression model that estimates dementia. Matched groups were not used for the later statistical analyses.

We quantified associations between BAI and neuropsychological test results among patients who had assessments available. Results were available for MoCA and MMSE. We analyzed BAI vs neuropsychological score associations by calculating Pearson correlation. Scores for MoCA and MMSE were pooled from all groups to maximize the strength of any possible underlying correlations.

To quantify the association of dementia and other covariates with increased BAI, we conducted a regression analysis with potential contributors of BAI, including age, sex, self-reported race/ethnicity (ie, White, Black, or Asian), obesity, insomnia, apnea-hypopnea index, periodic limb movement index, smoking, diabetes, anxiety disorder, psychotic disorder, mood disorder, cardiovascular disease, alcoholism, Epworth Sleepiness Scale, body mass index, and dementia. Dementia was binarized as belonging to dementia or nondementia groups. Sex and race/ethnicity were binarized into dummy variables. Continuous variables Epworth Sleepiness Scale, body mass index, and age were standardized using *z* score normalization. A linear regression model was built using all the covariates as independent variables to estimate BAI. Any missing data were imputed using means.

To identify which component features of BAI contribute to the associations between BAI and dementia group membership, we used the odds ratio (OR) of logistic regression models. Analyses were conducted using Python version 3.7 (Python Software Foundation). Cuzick tests used 1-tailed *P* values, and all other analyses used 2-tailed *P* values. Significance was set at .05. Data were analyzed from November 15, 2018, to June 24, 2020.

## Results

### Baseline Characteristics

Of 9834 sleep studies in the data set, we excluded patients younger than age 50 years and any with diagnoses of developmental delay, brain tumor, stroke, brain injury or trauma, or seizure before the sleep study, resulting in 4053 patients and 4690 EEGs excluded. Among 5144 included patients, the median (interquartile range) age was 54 (43-65) years and 3026 (59%) were men. Using criteria in the Table, we classified 81 patients (with 96 EEGs) into the dementia group, 44 patients (with 55 EEGs) into the MCI group, 1075 patients (with 1361 EEGs) into the symptomatic group, and 2336 patients (with 2799 EEGs) into the nondementia group.

### Association of BAI With Dementia

**Figure 1.**
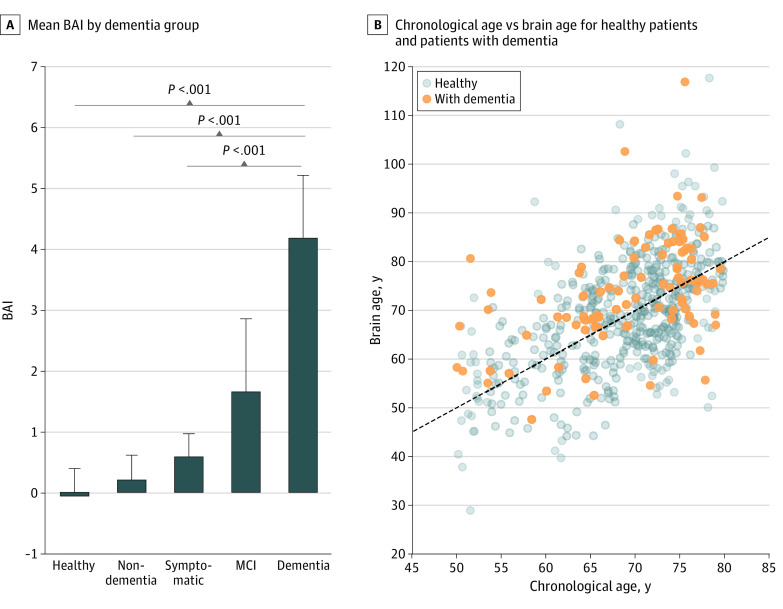
Brain Age Index (BAI) Across Dementia Groups The dashed line indicates BAI = 0; MCI, mild cognitive impairment.

We found that mean (SE) BAI increased from nondementia to dementia groups (nondementia: 0.20 [0.42]; symptomatic: 0.58 [0.41]; MCI: 1.65 [1.20]; dementia: 4.18 [1.02]; *P* < .001) (Figure 1). Pairwise comparisons indicated that the mean (SE) BAI for the dementia group (4.18 [1.02]) was significantly higher than that of the symptomatic group (0.58 [0.41]; *P* < .001), the nondementia group (0.20 [0.42]; *P* < .001), and the healthy group (−0.06 [0.45]; *P* < .001). The dementia group, compared with the nondementia group, had a significantly higher proportion of psychotic disorders (18 patients [19%] vs 53 patients [6%]; *P* < .001), mood disorders (61 patients [64%] vs 350 patients [40%]; *P* < .001), and anxiety disorders (49 patients [52%] vs 281 patients [31%]; *P* < .001); the prevalence of other conditions did not significantly differ between these groups (eTable 2 in the Supplement). Compared with other sleep macrostructure features, BAI had the highest OR for estimating dementia in a logistic regression model (OR, 1.44; 95% CI, 1.43-1.45; *P* < .001) followed by sleep fragmentation index (OR, .32; 95% CI, 1.31-1.32; *P* = .03) (eFigure 1 in the Supplement). Regardless of dementia status, men had higher mean (SE) BAI (3.05 [11.20] years) than women (−0.14 [10.95] years).

### Association of Neuropsychological Scores With BAI

**Figure 2.**
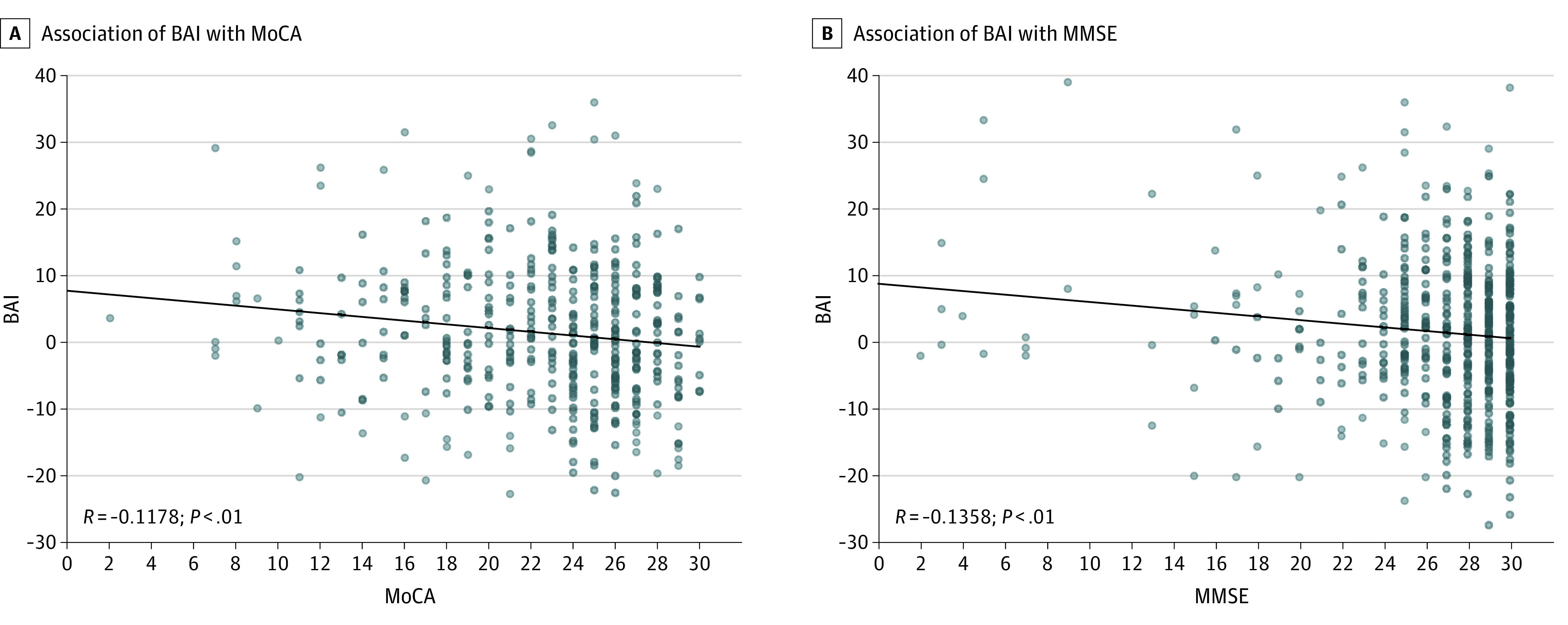
Associations of Neuropsychological Scores With Brain Age Index (BAI) MoCA indicates Montreal Cognitive Assessment; MMSE, Mini-Mental State Examination.

Associations of BAI with neuropsychological testing scores are illustrated in Figure 2. There were significant negative correlations between BAI and MoCA (*R* = −0.14; *P* = .006) and between BAI and MMSE (*R* = −0.12; *P* = .005).

### Regression Analysis of BAI Covariates

**Figure 3.**
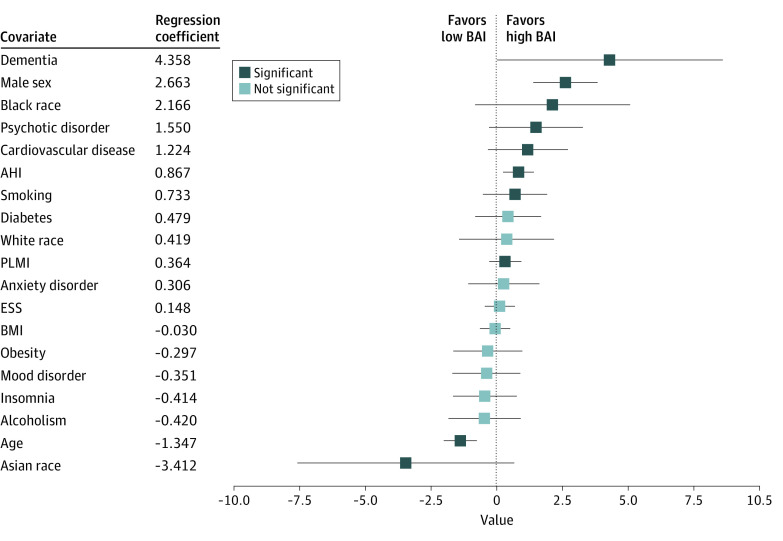
Regression Analysis of Potential Brain Age Index (BAI) Covariates With Dementia Squares indicate coefficients of covariates; whiskers, 95% CIs; AHI, apnea-hypopnea index; BMI, body mass index; ESS, Epworth Sleepiness Scale; and PLMI, periodic limb movement index.

Coefficients of covariates in a linear regression model that estimated BAI are shown in Figure 3. Dementia had the highest positive regression coefficient (SE) with BAI (4.36 [2.20]; *P* < .001), followed by nonmodifiable risk factors, such as male sex (2.67 [0.63]; *P* < .001) and Black race/ethnicity (2.17 [1.50]; *P* = .005). Other significant modifiable risk factors associated with accelerated brain aging include psychotic disorder (coefficient [SE], 1.55 [0.91]; *P* < .001), cardiovascular disease (coefficient [SE], 1.22 [0.79]; *P* = .002), apnea-hypopnea index (coefficient [SE], 0.87 [0.30]; *P* < .001), smoking (coefficient [SE], 0.73 [0.64]; *P* = .02), and periodic limb movement index (coefficient [SE], 0.36 [0.31]; *P* = .02). There were negative regression coefficients [SE] between BAI and age (−1.35 [0.32]; *P* < .001) and BAI and Asian race/ethnicity (−3.41 [2.10]; *P* = .002). Age had a nonzero but small coefficient (−1.35; *P* < .001), indicating some residual correlation of BAI with age despite our decorrelation procedure.

### Correlation Between Brain Age Features and Dementia

**Figure 4.**
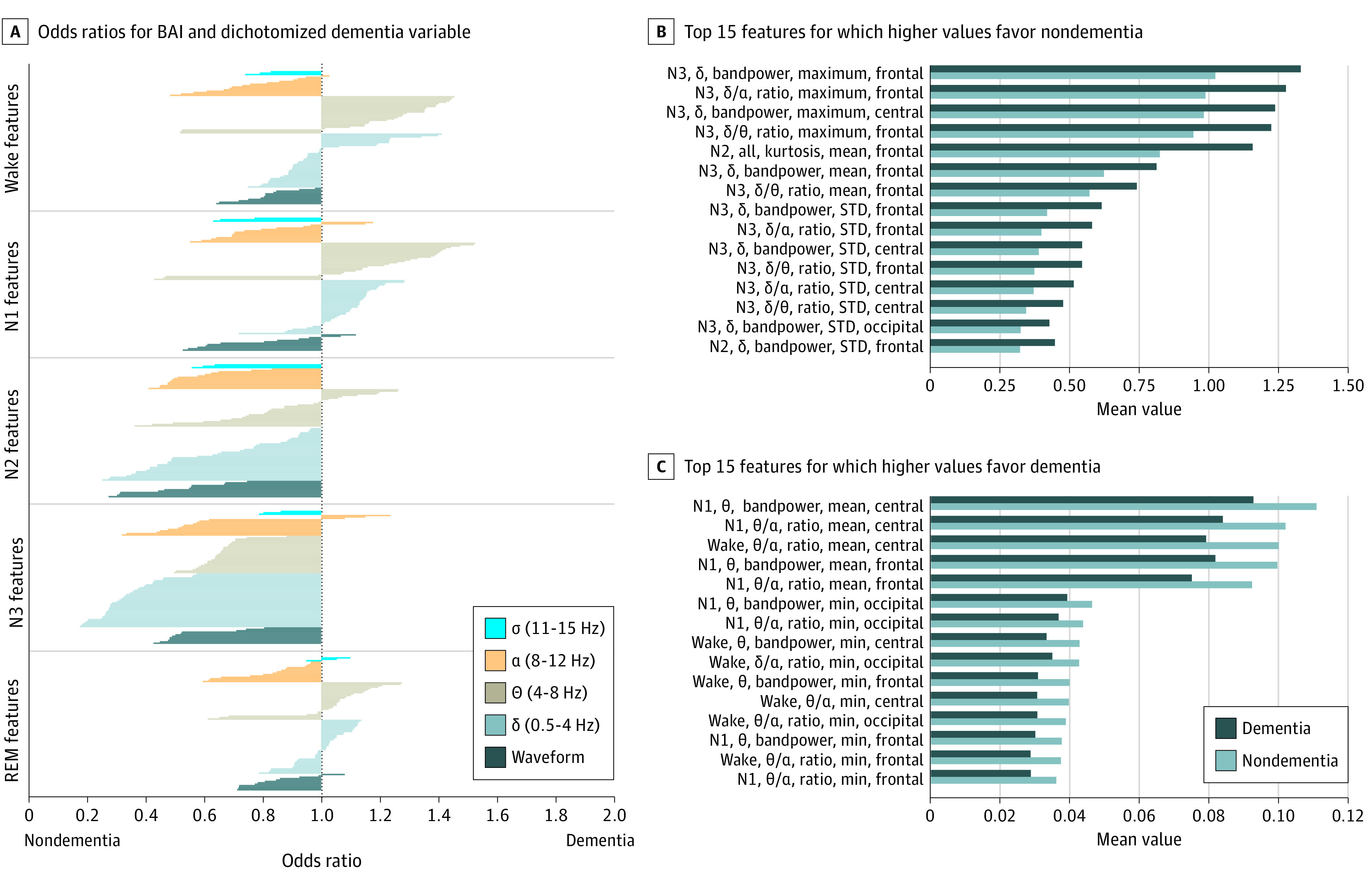
Association of Brain Age Index (BAI) Features and Dementia B and C, features names describe related sleep stage, frequency band, measure, statistic, and channel location of the feature in order. All correlations shown are statistically significant. REM indicates rapid eye movement; N, non–REM stage.

Of 480 BAI features, 54 had significant correlations with dementia and 213 had significant correlations with nondementia. The top 15 features of each are shown in Figure 4. Features related to δ activity in N2 and N3 sleep were more likely to be negatively correlated, while features related to θ and δ activity in W and N1 sleep were more likely to be positively correlated with dementia. Across all sleep stages, α oscillation features were consistently higher in the nondementia group.

### Representative Hypnograms and Spectrograms

Typical hypnograms and spectrograms from dementia, MCI, symptomatic, and nondementia groups are shown in eFigure 2 in the Supplement. Compared with healthy patients of the same age, the dementia example shows no visible spindles during N2, reduced α peak frequency (7.5 Hz), reduced δ and θ band power during N2 and N3, and low total power. The MCI example shows weak spindles, weaker δ power during N2 and N3 compared with symptomatic and nondementia groups, but more δ band power during N2 and N3 than the dementia group. The symptomatic example shows weaker spindle power compared with the nondementia example, but higher spindle power compared with the dementia group. The nondementia example shows high α peak frequency (11 Hz), strong δ power during N2 and N3, and strong spindle band power during N2.

## Discussion

The results of this cross-sectional study suggest that the sleep EEG BAI may be a promising biomarker associated with dementia across multiple levels of clinical severity. We found that patients with dementia had higher BAI than patients without dementia at the group level; BAI was correlated with neuropsychiatric scores; among all contributors of BAI tested, dementia had the strongest positive correlation; and among brain age features, stronger θ feature values in W and N1 stages were correlated with dementia, whereas stronger θ and δ features in N2 and N3 stages were correlated with nondementia.

The significantly increasing BAI from nondementia to dementia groups suggests dementia was associated with higher BAI. Patients with dementia had significantly higher BAIs than patients in the symptomatic, nondementia, and healthy groups at the group level. The significantly higher proportion of psychotic, mood, and anxiety disorders in the dementia group could be associated with symptoms caused by dementia. Patients with MCI did not have significantly different BAIs compared with the other groups, but the group mean was between the dementia and nondementia groups. One possible explanation might be that our MCI group included both stable and progressive MCI with different underlying causes. In a 1985 study by Cuzick,^17^ patients with progressive MCI who converted to Alzheimer disease (AD) and cognitively declined within 3 years of follow-up had significantly higher MRI-based brain age than patients with stable MCI. The high SE and the bimodal distribution suggested in the histogram of our MCI group supports this possibility (eFigure 3 in the Supplement). We used a matched case-control approach for the main analysis; this design is advantageous in that it avoids assumptions inherent in parametric regression approaches to confounder adjustment; it facilitates visualization of the group mean BAI values of the matched groups in bar plots; and it is flexible enough that we can define the precision of matching for age. However, matching restricts us to use only the matched subsets of the data and to adjust only for a few confounding variables at once; therefore, for the subsequent analyses we did not use matching. Because BAI had a significantly higher association with dementia compared with sleep macroarchitectural features derived from the hypnogram alone, it is clear that there were significant microarchitectural sleep differences between patients with dementia vs those without that accounted for BAI above and beyond differences derived from conventional sleep scoring.

We found that BAI was correlated with MoCA and MMSE, with the correlation being stronger in MoCA compared with MMSE. A 2018 study^18^ demonstrated MRI-derived brain age was significantly correlated with traditional AD screening tools, including MMSE. While the diagnostic performance of MMSE for dementia is good, MMSE does worse at detecting MCI (sensitivity, 0.62) compared with MoCA (sensitivity, 0.89).^19^ The stronger correlation of MoCA scores with MCI may contribute to the stronger correlation that we observed between MoCA and BAI.

Regression analysis of potential BAI covariates suggest dementia was the strongest contributor associated with BAI compared with other conditions, such as psychiatric and cardiovascular disease. Male sex is the second greatest contributor; with or without dementia, men had higher mean (SE) BAI (3.05 [11.20] years) than women (−0.14 [10.95] years). This could reflect that men experience more brain atrophy with aging.^20^ Racial/ethnic differences were noted in our analysis, with lower BAI for Asian patients and higher BAI for Black patients. These findings appear consistent with prior epidemiological studies reporting that Black individuals have the highest and Asian individuals the lowest risk among racial/ethnic groups in the US.^21^ However, our observations should be considered preliminary given sample size limitations. Both apnea-hypopnea index and periodic limb movement index have been associated with decreased cognitive performance^22,23,24^ and were also associated with higher BAI.

Our results suggest patients with dementia had less robust δ oscillations during N2 and N3 sleep. The nondementia group had higher δ and θ features in N2 and N3 compared with the dementia group. These findings align with literature suggesting that robust slow-wave sleep features are associated with preserved cognitive function^25^ and memory consolidation.^26^ Accumulating evidence suggests that people with disturbed sleep have a higher risk of cognitive decline and AD dementia,^27^ potentially owing to lack of deep (ie, N3) sleep, in which δ oscillations predominate. A suggested mechanism is that δ oscillation during non-REM sleep are coupled to oscillations in cerebrospinal fluid flow, which promote clearance of amyloid deposits and plaques, a hallmark of AD.^28^

The dementia group had higher θ and δ features in W and N1 stages compared with the nondementia group. Penttila et al^29^ found significant increases in θ power in mild AD and a marked increase in δ power in severe AD, and Chiaramonti et al^30^ reported similar findings. Given the negative correlation of α power with dementia, the decrease in α power could indicate atrophy of the hippocampus.^31^ The higher θ/α ratio features in patients with dementia is supported by a 2009 study^32^ that found lower α/slow-wave power ratios in patients with AD. The increased θ/α ratio is also consistent with several resting-state EEG findings, for example, increased θ/α ratio correlates with lower cognition scores in 20 elderly adults,^33^ and θ/α ratio was higher patients in AD compared with individuals without AD with the same age and sex.^34^ This is also associated with a decrease in α peak frequency with aging. The α peak frequency is approximately 10 Hz for young adults and gradually decreases to 8 or 7 Hz in elderly individuals.^35^

### Limitations

Our study has some important limitations. First, sleep staging was manual. Given that the interrater agreement for the American Academy of Sleep Medicine standard is typically 82%,^36^ an automated method might be more accurate. However, automated staging algorithms are trained on expert scoring,^37^ so expert-based sleep staging is currently the closest representation of ground truth available. Second, it is theoretically possible that sleep staging is less reliable in patients with dementia. To investigate this possibility, we investigated the sleep stage distribution across groups. Reassuringly, we found the well-known decreasing trend of mean (SE) REM from the nondementia (15.80% [0.54%]) to the dementia group (12.86% [0.10%]) (eFigure 4 in the Supplement), consistent with research documenting impaired REM in dementia.^38^ Third, noise in the EEG may confound BAI estimates. A 2020 preprint^39^ reported relatively high night-to-night variability of BAI within individuals. This is not a critical issue for our study, given the focus on group-level outcomes. However, future work should aim to improve the signal-to-noise ratio of BAI at an individual level. Fourth, BAI is derived from an algorithm optimized to estimate age instead of dementia. While BAI is correlated with dementia, dementia-related and age-related network changes of the brain differ. Alzheimer disease preferentially degrades sensorimotor networks and within-network connections over internetwork connections, while aging within reference ranges decreases default network connectivity in a more diffused pattern.^40^ Optimizing algorithms to detect these network-specific changes might provide even more powerful biomarkers to detect dementia.

## Conclusions

This cross-sectional study found that the sleep EEG-based BAI showed potential as a biomarker associated with deviation from healthy brain aging, including processes leading to dementia. Sleep EEG is increasingly accessible in non–sleep laboratory environments, including the home, and using wearable technologies, such as headbands and dry EEG electrodes. Thus, it is feasible to obtain multiple nights of EEG. Clinical utility at an individual level requires further development and testing, including using wearable technologies and multiple nights of data.

## Notes

Supplement. **eTable 1.** Summary of Characteristics of the Sleep Data Set **eTable 2.** Comparison of Phenotypes Between Matched Dementia and Nondementia Groups **eAppendix.** Calculation of the Brain Age Index: Technical Details **eFigure 1.** Associations of Brain Age Index and Sleep Macrostructure With Dementia **eFigure 2.** Hypnogram and Spectrogram of Typical Studies From Each Dementia Group **eFigure 3.** Histogram Showing Distributions of Brain Age Index for Healthy Group vs Dementia, Mild Cognitive Impairment, Symptomatic, and Nondementia Groups After Matching **eFigure 4.** Stacked Bar Plot Shows Distribution of Sleep Stages Across Groups
