# Supplementary material for: Association of Sleep Electroencephalography-Based Brain Age Index With Dementia
Source: JAMA Netw Open. 2020 Sep 28;3(9):e2017357. doi: 10.1001/jamanetworkopen.2020.17357 (PMC7522697; doi:10.1001/jamanetworkopen.2020.17357)
Supplement: Supplement. — eTable 1. Summary of Characteristics of the Sleep Data Set eTable 2. Comparison of Phenotypes Between Matched Dementia and Nondementia Groups eAppendix. Calculation of the Brain Age Index: Technical Details eFigure 1. Associations of Brain Age Index and Sleep Macrostructure With Dementia eFigure 2. Hypnogram and Spectrogram of Typical Studies From Each Dementia Group eFigure 3. Histogram Showing Distributions of Brain Age Index for Healthy Group vs Dementia, Mild Cognitive Impairment, Symptomatic, and Nondementia Groups After Matching eFigure 4. Stacked Bar Plot Shows Distribution of Sleep Stages Across Groups [file jamanetwopen-e2017357-s001.pdf]

Supplementary Online Content

Ye E, Sun H, Leone MJ, et al. Association of sleep electroencephalography-based brain age index with dementia. *JAMA Netw Open*. 2020;3(9):e2017357. doi:10.1001/jamanetworkopen.2020.17357

eTable 1. Summary of Characteristics of the Sleep Data Set

eTable 2. Comparison of Phenotypes Between Matched Dementia and Nondementia Groups

eAppendix. Calculation of the Brain Age Index: Technical Details

eFigure 1. Associations of Brain Age Index and Sleep Macrostructure With Dementia

eFigure 2. Hypnogram and Spectrogram of Typical Studies From Each Dementia Group

eFigure 3. Histogram Showing Distributions of Brain Age Index for Healthy Group vs Dementia, Mild Cognitive Impairment, Symptomatic, and Nondementia Groups After Matching

eFigure 4. Stacked Bar Plot Shows Distribution of Sleep Stages Across Groups

This supplementary material has been provided by the authors to give readers additional information about their work.

© 2020 Ye E et al. *JAMA Network Open.*

eTable 1. Summary of Characteristics of the Sleep Data Set

Values of age, BMI, and ESS are shown as medians with interquartile ranges. and all other values are shown as counts.

**Characteristics**	**Value**
Number of PSGs	9,834
Number of patients	8,207
Age	54 (43-65)
BMI (kg/m^2)	31 (27-36)
Epworth Sleepiness Scale (ESS)	8 (4-12)
Sex	
Female	4026 (41%)
Male	5732 (59%)
Ethnicity	
Asian	291 (3%)
White	7542 (76%)
Black	640 (6%)
Hispanic	442 (4%)
Other/unknown	1030 (10%)
Type of Test	
Diagnostic	4799 (48%)
All night CPAP	2539 (25%)
Split night	2607 (26%)
Apnea-Hypopnea Index (AHI, events / hour)	
Normal (AHI < 5)	3738 (38%)
Mild (5 ≤ AHI < 15)	2854 (29%)
Moderate (15 ≤ AHI < 30)	1,917 (19%)
Severe (AHI ≥ 30)	1046 (11%)

© 2020 Ye E et al. *JAMA Network Open.*

eTable 2. Comparison of Phenotypes Between Matched Dementia and Nondementia Groups

P-values of two-samples proportions z-test are shown.

**Phenotypes**	**Dementia**	**Non-Dementia**	**P-value**
Smoking	33 (35%)	310 (34%)	0.99
Alcoholic	27 (28%)	330 (37%)	0.10
Insomnia	29 (30%)	257 (29%)	0.72
Mood disorders	61 (64%)	350 (40%)	**< 0.001**
Psychotic disorders	18 (19%)	53 (6%)	**< 0.001**
Anxiety disorders	49 (52%)	281 (31%)	**< 0.001**
Diabetes	37 (39%)	432 (48%)	0.08
Obesity	38 (40%)	414 (46%)	0.24
Cardiovascular diseases	67 (71%)	707 (79%)	0.05
Sleep Apnea (AHI > 5)	59 (62%)	639 (72%)	0.05

© 2020 Ye E et al. *JAMA Network Open.*

eAppendix. Calculation of the Brain Age Index: Technical Details

The Brain Age Model is a generalized linear model (GLM) that uses a softplus function as the link function.

We used the same preprocessing for the EEG and brain age computation as in Sun et al[7]. Briefly, the 6 EEG channels (F3-M2, F4-M1, C3-M2, C4-M1, O1-M2, O2-M1) were notch-filtered at 60Hz and then bandpass filtered at 0.5Hz to 20Hz. All 30-second epochs with maximum absolute amplitude greater than 500uV were removed. Epochs containing >2 seconds of flat signal (standard deviation less than 0.2uV) were also removed. For each recording, the amplitude of each channel was normalized to the median 0 and unit interquartile range.

EEGs were selected from a group of approximately 10,000 PSGs. EEGs were selected were those without errors in time stamps, that had available sleep stage annotations, and at least 100 artifact-free 30-second epochs. The total number of studies meeting these criteria was 9834. The sampling frequency for EEG siganls was 200 Hz. EEGs each had 6-channels (see below), as is conventional in polysomnography recordings. Seven sleep technicians annotated the EEGs, with one technician per PSG. Sleep staging was subsequently followed by review and revision as needed by a licensed sleep physician.

As defined in Table S4 in Sun et al[7], features were extracted from overnight sleep EEG recordings and included line lengths and kurtosis from each of the 6 channels (F3-M2,F4-M1, C3-M2,C4-M1,O1-M2,O2-M1) and delta (1-4 Hz), theta (4-8 Hz), alpha (8-12 Hz), and sigma (12-16 Hz) band powers (and their ratios) and spectral kurtosis from the 3 pairs of laterally averaged channels. Sample entropy features were excluded due to high computational burden. These features were then averaged across all 30-second epochs for each sleep stage, yielding 96 x 5 = 480 features for each EEG recording. The regression objective was to minimize the mean squared error between CA and predicted BA. The model was penalized by the absolute value of the correlation between CA and BAI and the weight of the penalty, lambda, was a tuned based on a random subset of 300 EEGs from the training set of 1343 EEGs (internal validation set) to protect against overfitting. Once the optimal lambda is determined, the validation set is combined with the rest of the training set, and the model is retrained using the optimal lambda. For subjects with missing sleep stages, features were imputed with the average of the 10 closest subjects in terms of the Euclidean distance of features from other sleep stages. Multiple studies have observed a systematic bias in brain age estimates, where brain age tends to be overestimated in younger subjects and underestimated in older subjects[7,13,14]. The reason for this bias of the linear model framework may be due to the non-Gaussian distribution of age caused by the strict age group cutoffs (18 to 80 years old)[15]. To correct for this bias, we first computed the average BAI of each age groups of 18-20, 20-25, 25-30, ..., 75-80 years within the training set of healthy subjects to obtain the age-dependent adjustment values for each group. Then, we de-biased the predicted BAI by subtracting with these adjustment values so that the scatter of BAI has zero means across all age groups. While many works followed a residualization rational to adjust for the bias[15,16], we utilized a nonparametric approach that alleviated the linear assumption and therefore offered more robustness against model misspecification.

© 2020 Ye E et al. *JAMA Network Open.*

eFigure 1. Associations of Brain Age Index and Sleep Macrostructure With Dementia

Feature abbreviations include percentages of Stage N1 (N1%), Stage N2 (N2%), Stage N3 (N3%), and Stage R (REM%), as well as Total Sleep Time (TST) and Sleep Fragmentation Index (SFI). Plot shows odds ratio, indicated by circles, and their 95% confidence intervals, indicated by bars. Filled circles indicate statistical significance.

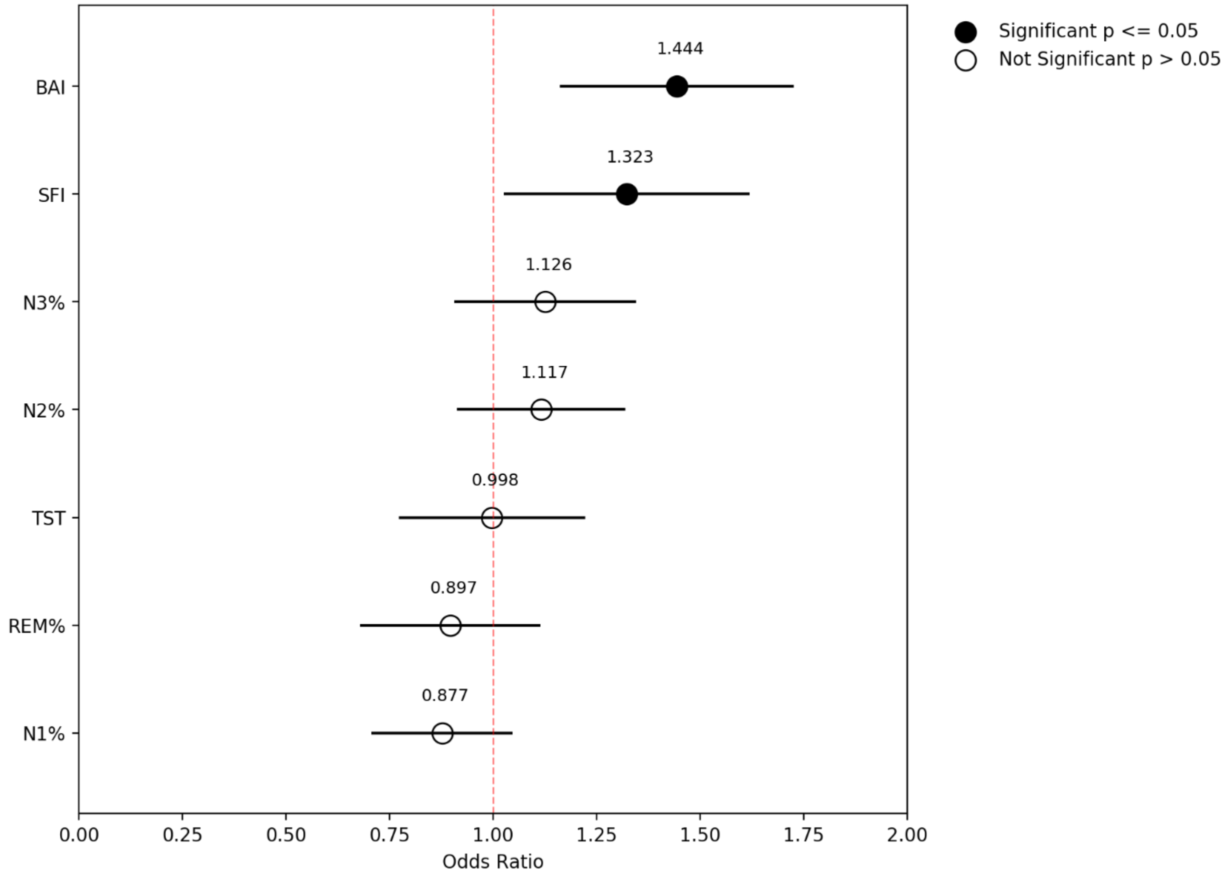

© 2020 Ye E et al. *JAMA Network Open.*

eFigure 2. Hypnogram and Spectrogram of Typical Studies From Each Dementia Group

(A) (B) (C) and (D) shows the hypnograM and spectrograM for Dementia, MCI, symptomatic, and non-dementia groups respectively. Sleep Stages included are as follows: Stage W (Wakefulness), Stage N1 (NREM 1), Stage N2 (NREM 2), Stage N3 (NREM 3), and Stage R (REM). On the right of each spectrograM, red brackets mark the alpha peak frequency band and blue brackets mark the spindle bands.

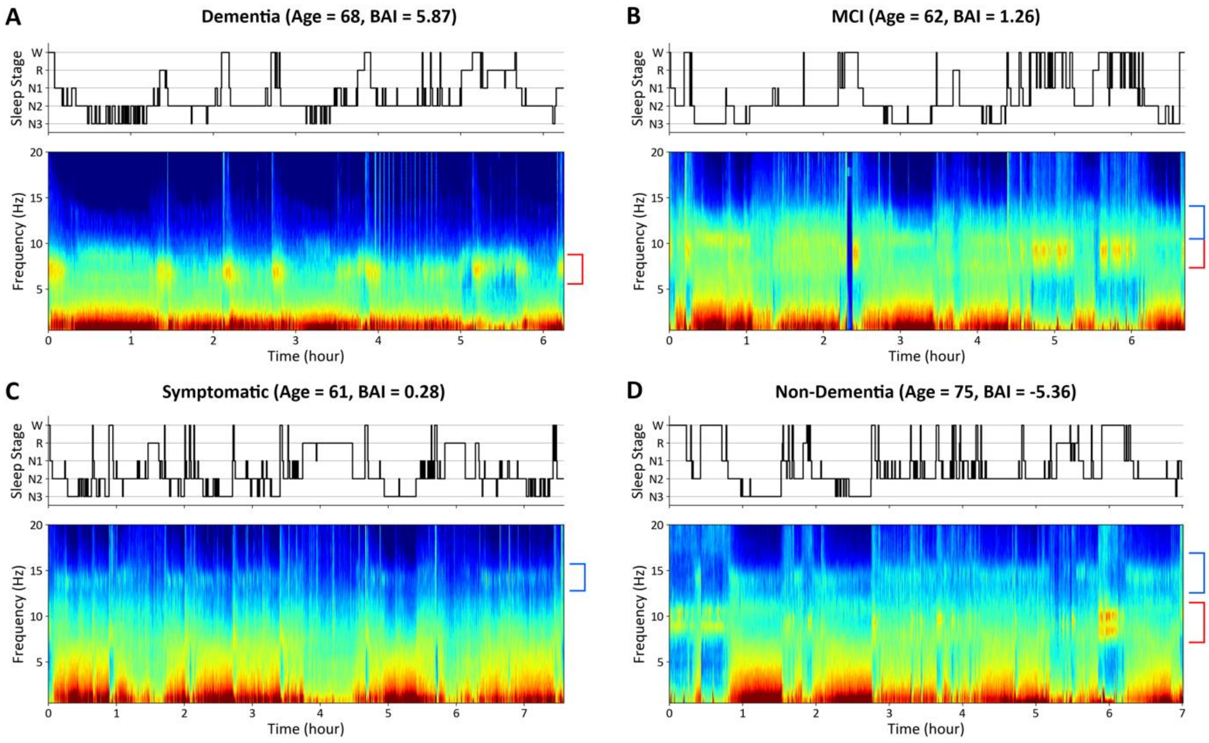

© 2020 Ye E et al. *JAMA Network Open.*

eFigure 3. Histogram Showing Distributions of Brain Age Index for Healthy Group vs Dementia, Mild Cognitive Impairment, Symptomatic, and Nondementia Groups After Matching

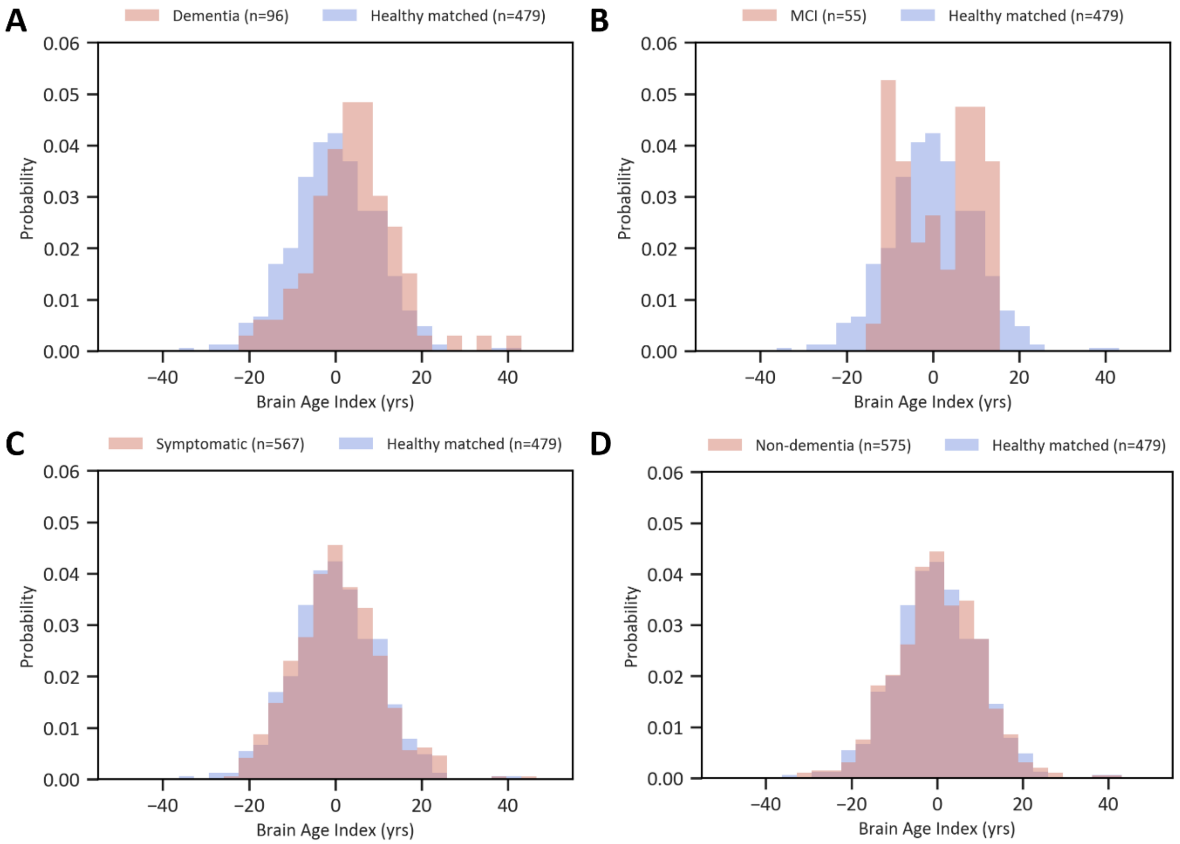

© 2020 Ye E et al. *JAMA Network Open.*

eFigure 4. Stacked Bar Plot Shows Distribution of Sleep Stages Across Groups

X-axis shows percent sleep stage of total sleep time.

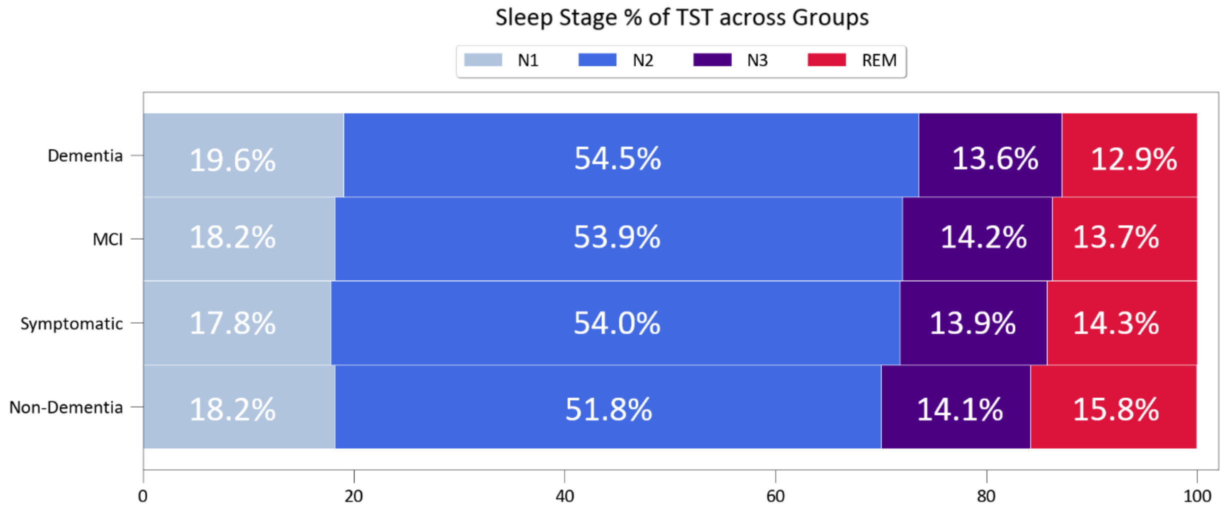

© 2020 Ye E et al. *JAMA Network Open.*
